# Dynamics of hepatic gene expression and serum cytokine profiles in single and double-hit burn and sepsis animal models

**DOI:** 10.1016/j.dib.2015.02.018

**Published:** 2015-03-11

**Authors:** Rohit Rao, Mehmet A. Orman, Francois Berthiaume, Ioannis P. Androulakis

**Affiliations:** a Department of Chemical and Biochemical Engineering, Rutgers, The State University of New Jersey, Piscataway, NJ 08854, USA; b Chemical and Biological Engineering Department, Princeton University, Princeton, NJ 08544, USA; c Department of Biomedical Engineering, Rutgers, The State University of New Jersey, Piscataway, NJ 08854, USA

**Keywords:** Burn, Cecal ligation and puncture, Chemokines, Cytokines, Hepatic gene expression, Inflammatory response, Microarray analysis

## Abstract

We simulate the pathophysiology of severe burn trauma and burn-induced sepsis, using rat models of experimental burn injury and cecal ligation and puncture (CLP) either individually (singe-hit model) or in combination (double-hit model). The experimental burn injury simulates a systemic but sterile pro-inflammatory response, while the CLP simulates the effect of polymicrobial sepsis. Given the liver׳s central role in mediating the host immune response and onset of hypermetabolism after burn injury, elucidating the alterations in hepatic gene expression in response to injury can lead to a better understanding of the regulation of the inflammatory response, whereas circulating cytokine protein expression, reflects key systemic inflammatory mediators. In this article, we present both the hepatic gene expression and circulating cytokine/chemokine protein expression data for the above-mentioned experimental model to gain insights into the temporal dynamics of the inflammatory and hypermetabolic response following burn and septic injury. This data article supports results discussed in research articles (Yang et al., 2012 [1,4]; Mattick et al. 2012, 2013 [2,3]; Nguyen et al., 2014 [5]; Orman et al., 2011, 2012 [6–8]).

**Specifications table**

**Table:** 

Subject area	Biology
More specific subject area	Inflammation, sepsis, burns, bioinformatics
Type of data	Gene expression data (.CEL files) and serum cytokine profiles (Excel tables)
How data was acquired	mRNA expression using Affymetrix Rat Genome 230 2.0 Array Microarrays, Cytokine expression using MILLIPLEX Rat Cytokine/Chemokine Panel
Data format	Raw (.CEL files) gene expression data. Analyzed data for serum cytokine profiles
Experimental factors	Collected liver samples were flash frozen for off-line microarray analysis
Experimental features	Combination of non-lethal rat models of burn injury and cecal ligation and puncture
Data source location	599 Taylor Road, Piscataway, New Jersey,08854, U.S.A.
Data accessibility	Data is presented along with this article

## Value of the data

• We present a uniquely comprehensive dataset describing the short-term (up to 24 h) and long-term (up to 10 days) responses of hepatic gene expression and circulating cytokine dynamics in a combination of single- and double-hit animal models of experimental burn and sepsis (CLP). By obtaining short-term and long-term data both the immediate onset as well as the evolution of the inflammatory response to injury can be studied.
• Temporal expression profiles of rat hepatic mRNA are obtained for the various injury models by microarray analysis using a Rat Genome 230 2.0 Array (GeneChip, Affymetrix, Santa Clara, CA, USA) that consists of 31099 probe sets analyzing over 28,000 genes. Further information regarding the array is available at the manufacturer׳s website (http://www.affymetrix.com) [1–5].
• Temporal variation in serum concentrations of a panel of 23 serum cytokines and chemokines is analyzed at the protein (peptide) level [6–8]. The selected panel includes both pro-inflammatory and anti-inflammatory cytokines and chemokines whose expression is commonly altered after burn injury and sepsis.
• The short-term hepatic gene expression response in single-hit experimental burn and sepsis animal models is characterized [1,4].
• The long-term gene expression dynamics in the single-hit animal model of sepsis is determined [2].
• The effect of burn priming on metabolic and immune gene expression in the rat liver is determined in the animal model of sepsis [3].
• The dynamics of the early and long-term response in serum cytokine profiles is determined in the single-hit burn model and the single-hit sepsis model as well as in the double-hit burn and sepsis model [7,6,8].
• The data can be used to identify patterns of circadian gene expression in the homeostatic rat liver [5].

## 1 Methods

6–7 week old male Sprague–Dawley rats weighing between 150 and 200 g were used for this study. Animals were housed in a temperature-controlled environment (maintained at 25 °C) with a 12-h light–dark cycle and provided water and standard chow ad libitum. All experimental procedures were in accordance with the National Research Council guidelines and approved by the Rutgers University Animal Care and Facilities Committee.

## 2 Burn injury

A hypermetabolic response was induced by administering a full-thickness dorsal-skin burn to an area corresponding to 20% of the total body surface area (TBSA). Rats were randomly assigned to either a Burn group or a control Shamburn group. Rats were anesthetized by intraperitoneal injection of ketamine (80–100 mg/kg) and xylazine (12–10 mg/kg), and all hair was removed from the dorsal abdominal area using electric clippers. In the experimental Burn group, the animal׳s back was immersed in water at 100 °C for 10 s to produce a full-thickness scald injury covering 20% TBSA. An intraperitoneal injection of saline solution (50 ml/kg) was used to resuscitate the rats immediately after administering the burn injury. Rats sustaining the burn injury had a 100% survival rate with no evidence of systemic hypo-perfusion and no significant alterations in feeding patterns. Rats in the negative control, Shamburn group, were treated identically but were immersed in lukewarm water maintained at 37 °C. Rats were caged individually after burn or shamburn procedures and given standard rat chow and water ad libitum until sacrifice. Consistent with other studies with this full thickness burn model, no post-burn analgesics were administered since the nerve endings in the skin are destroyed and the skin becomes insensate. Furthermore, after animals woke up, they ate, drank and moved freely around the cage, responded to external stimuli, and did not show clinical signs of pain or distress. Animal body weights were monitored daily and found to increase at the same rate in both groups [1].

## 3 Cecal ligation and puncture

48 h after receiving burn or shamburn treatments, rats were anesthetized as described above, followed by the subcutaneous administration of analgesics, buprenorphrine (0.01–0.05 mg/kg) and bupivicaine (0.125–0.25%). A 2 cm midline incision was made in the rat abdominal cavity and the cecum of the animal was exposed and ligated just below the ileocecal valve so there was no intestinal obstruction. Care was taken not to ligate the cecal branch of the ileocecal artery, thus preserving viability of the cecum itself, in order to increase the survival rate. The cecum was punctured four times (through only one surface of cecum at each instance of a puncture) with a 20-ga needle and replaced in the peritoneum. The abdominal incision was then sutured in layers using interrupted monofilament sutures. The animal was subsequently resuscitated with saline solution administered intraperitonially (10 mL/kg). The Sham-CLP (SCLP) procedure, which is the control of CLP, involved treating animals identically, however, without administering cecal ligation and puncture [7].

## 4 Experimental design

Our animal model simulates a burn-induced hypermetabolic, immunosuppressive response followed by induction of polymicrobial sepsis, via CLP. A total of 6 animal cohorts were analyzed temporally. Three animals were sacrificed at each time point so as to have a balance between accuracy of the experimental data and the practical considerations of having a large number of experimental groups being analyzed at appropriate time intervals. The Shamburn group served as the negative control for the Burn, Shamburn-SCLP and the Shamburn-CLP groups. Furthermore, the Shamburn-SCLP group was the control for the Shamburn-CLP group, while the Burn-SCLP group was the control for the Burn-CLP group. Specifically, the six groups were studied as follows:

(1) Shamburn (CEL file: Shamburn): Animals were sacrificed right before shamburn (time *t*=0 corresponding to 9 a.m.) and then at 2, 4, 8, 16 and 24 h post shamburn treatment. The CEL files contain the following information: Affymetrix probe IDs, followed by triplicate expression values at above mentioned sample collection points.
(2) Burn (CEL file: Burn): Animals were sacrificed right before the burn injury was administered (time *t*=0; 9 a.m.) and then 2, 4, 8, 16 and 24 h post burn injury. The CEL files contain the following information: Affymetrix probe IDs, followed by triplicate expression values at above mentioned sample collection points
(3) Shamburn-SCLP (CEL file: S-SCLP): Animals received SCLP treatment 48 h post shamburn treatment. Animals were sacrificed right before SCLP treatment (time *t*=0, 9:00 a.m.) and then at 2, 4, 8, 12, 16, 20, 24, 48, 120 and 192 h post SCLP. Please note, the sample collection point *t*=0 corresponds to 48 h post shamburn. The CEL files contain the following information: Affymetrix probe IDs, followed by triplicate expression values at above mentioned sample collection points.
(4) Shamburn-CLP (CEL file: S-CLP): Animals received CLP treatment 48 h post shamburn treatment. Animals were sacrificed right before CLP treatment (time *t*=0, 9:00 a.m.) and then at 2, 4, 8, 12, 16, 20, 24, 48, 120 and 192 h post CLP. Please note, the sample collection point *t*=0 corresponds to 48 h post shamburn. The CEL files contain the following information: Affymetrix probe IDs, followed by triplicate expression values at above mentioned sample collection points.
(5) Burn-SCLP (CEL file: B-SCLP): Animals received SCLP treatment 48 h post burn injury. Animals were sacrificed right before SCLP treatment (time *t*=0, 9:00 a.m.) and then at 2, 4, 8, 12, 16, 20, 24, 48, 120 and 192 h post SCLP. Please note, the sample collection point *t*=0 corresponds to 48 h post burn injury. The CEL files contain the following information: Affymetrix probe IDs, followed by triplicate expression values at above mentioned sample collection points.
(6) Burn-CLP (CEL file: B-CLP): Animals received CLP treatment 48 h post burn injury. Animals were sacrificed right before CLP treatment (time *t*=0, 9:00 a.m.) and then at 2, 4, 8, 12, 16, 20, 24, 48, 120 and 192 h post CLP. Please note, the sample collection point *t*=0 corresponds to 48 h post burn injury. The CEL files contain the following information: Affymetrix probe IDs, followed by triplicate expression values at above mentioned sample collection points.

Euthanasia is by exsanguination during retrieval of liver tissue under general anesthesia. These methods are consistent with recommendations of the Panel on Euthanasia of the American Veterinary Medical Association.

## 5 Microarray analysis

After sacrificing the animals, liver tissues were collected and flash frozen for offline microarray analysis (*n*=3 per time point per group). The tissues were lysed and homogenized using Trizol, and the RNAs were further purified and treated with DNase using RNeasy columns (Qiagen, Valencia, CA, USA). Then cRNAs prepared from the RNAs of liver tissues using protocols provided by Affymetrix were utilized to hybridize Rat Genome 230 2.0 Array (GeneChip, Affymetrix, Santa Clara, CA, USA) comprised of 31099 probe sets. Two microarray batches were used to analyze the data. The Shamburn and Burn groups were analyzed in the first batch, while the remaining groups were analyzed in the second batch.

Genome expression data can be extracted from the.CEL files using DNA chip analyzer (dChip) software with invariant-set normalization and a perfect-match model [9].

## 6 Cytokine analysis

Animals were sacrificed and anesthetized at the above mentioned time points. Heparinized catheters were used to collect blood samples from the rat vena cava. The collected samples were stored on ice until further use for serum preparation. Serum was prepared by centrifuging the samples at 4500 rpm for 3 min at 4 °C and stored at −80 °C until analyzed. A MILLIPLEX MAP (multi-analyte panels) Rat Cytokine/Chemokine Panel (Millipore) was used according to manufacturer׳s guidelines in order to simultaneously quantify concentrations of 23 different cytokines and chemokines at protein (or peptide) level (Eotaxin, G-CSF, GM-CSF, GRO/KC, IFN-γ, IL-10, IL-12 (p70), IL-13, IL-17, IL-18, IL-1α, IL-1β, IL-2, IL-4, IL-5, IL-6, IP-10, Leptin, MCP-1, MIP-1α, RANTES, TNF-α, VEGF).

In summary, we present a comprehensive account of temporal liver-specific transcriptional dynamics and temporal circulating cytokine/chemokine dynamics in a rat model of burn/sepsis (single and combined injury). However, much like any in vivo study variability in responses is a key complicating factor. Despite the fact that the burn and CLP models used are considered the “gold standard”, the systemic nature of the burn injury and the non-specific character of the polymicrobial infection induced by CLP complicates the analysis. Nevertheless, this weakness should be perceived as the challenge when dealing with animal models aiming at reproducing physiological responses to trauma. Finally, it is well established that cytokine measurements tend to exhibit higher variability, which was apparent in our studies.
